# Editorial: Can population health be personalized? Estonia and Finland as examples

**DOI:** 10.3389/fgene.2022.1051085

**Published:** 2022-10-24

**Authors:** Helena Kääriäinen, Andres Metspalu, Markus Perola

**Affiliations:** ^1^ Department of Public Health and Welfare, Finnish Institute for Health and Welfare, Helsinki, Finland; ^2^ Estonian Genome Centre, Institute of Genomics, University of Tartu, Tartu, Estonia; ^3^ Research Program for Clinical and Molecular Metabolism, Faculty of Medicine, University of Helsinki, Helsinki, Finland

**Keywords:** biobanks, population health, genomics, early diagnostics, health promotion

Healthcare systems in the developed countries are facing growing challenges due to rising costs of treatments, aging populations and shortage of health care personnel. All this accentuates the need to develop effective prevention and early diagnostics for common chronic diseases. One of the suggested solutions has been wider use of genomics. At present, genomic medicine mainly serves to improve diagnostics of rare diseases and precision of oncological medications but new developments seem to offer hope for possibilities to implement genomics to improve population health. One solution might be screening for risk individuals in biobanks and approaching them to offer prevention and treatment. Both Estonia and Finland belong to the pioneers in national biobanking, and legislation as well as collecting biobank samples is well advanced in both countries making them excellent environments to start implementation of genomics to population health.

In Estonia, Human Gene Research Act was approved 2001 and the Estonian Genome Center was established. Today the Estonian Biobank has samples from over 200.000 people (about 20% of the adult population), and rich health related and environmental data has been collected from all of them. All samples are genotyped with Global Screening Array and data are imputed against the Estonian whole genome sequence reference. There are plans for implementing the data to personalized medicine/prevention in healthcare as a general service starting 2023.

Finnish public entities, universities and research institutes have for decades collected large studies with biological samples, including DNA, and the collection is on-going facilitated by the nation-wide project FinnGen (www.finngen.fi). These population-based or disease-oriented collections have been biobanked under the Finnish Biobank Act. This resource, samples and genotype data of soon more than 500.000 individuals can be linked using the unique personal identification code to the nation-wide health registers for scientific studies under the Finnish law for the secondary use of data.

The aim of this Research Topic is to describe some of the first experiences of piloting and implementing use of genomic data at the level of the population healthcare and to describe processes and tools for population-wide use of genomics.

Wahlfors et al. draft in their paper the necessary steps towards the possible large-scale implementation of genomic medicine in population healthcare in Finland. Among the national actions needed are, for instance, providing scientific evidence for the utility, developing the legal framework and necessary infrastructures, and evaluating the potential health-economic impact of implementing precision healthcare in Finland. The authors stress the importance of public engagement and training genomic literacy skills among various target groups.

As reporting individual results from biobanks to each donor in separate counselling sessions appears very tedious, Marjonen et al. have piloted the use of a secure web portal for communicating polygenic risk score (PRS) results combined with clinical risk factors. They report results from the P5-study which suggest that this was a feasible way to report genetic risk information and estimated disease risk comprehensively so that not only citizens themselves could take advantage of it, but also it provided guidelines for health care professionals.

In the first report of the planned follow-up studies of the P5 participants, Halmesvaara et al. compared the results from the randomized controlled setting of P5, where the experimental group received risk estimates based on traditional and polygenic risk factors and the control group based solely on traditional risk factors. Based on follow-up questionnaires, they found no evidence that adding the polygenic risk to complement the traditional risk factors would induce any substantive psychosocial harm to the recipients.

In Estonia, instead of plans and piloting, the first implementations of using biobank data for improving population health have already been performed in research setting. In this Research Topic, Jürgens et al. have employed “genomics first” approach to detect carriers of 11 breast cancer–related genes in the Estonian Biobank donors and re-contacted them to discuss personalized prevention measures. Of the carriers detected, only one-third would have been eligible for clinical screening according to the current criteria which suggests that using existing genomic data from biobanks can be very valuable for early diagnostics of breast cancer.

Sarv et al. calculated the birth prevalence of spinal muscular atrophy (SMA) in Estonia which they considered as an effective starting point for adding SMA to the newborn screening program. As SMA usually progressed quickly and as the current treatment options are expensive, treatment should be started early to achieve best possible effect. On the other hand, the authors discussed the possibility of an Estonian carrier screening program in collaboration with Estonian Biobank. The partners of biobank donors identified as SMA carriers could be offered carrier testing. This approach could help early detection of newborns with SMA or offer a possibility to family planning.

If genomics will be widely used at the level of population health, a lot of training of health care professionals, especially public health nurses, is needed. Laaksonen et al. present the process of defining core genomic competencies for nursing and the pioneering education program of public health nurses for applying genomics in preventive healthcare. This training program, partially funded by Finnish Ministry of Education and Culture, introduces a new type of expertise in healthcare.

Martikainen et al. used Type 2 diabetes (T2D) as an example when assessing the cost-utility of adding PRS to the overall risk assessment followed by a lifestyle intervention and medical therapy if needed. For a cost-utility analysis, an individual-level state-transition model with probabilistic sensitivity analysis was constructed. The authors concluded that the PRS provides moderate additional value in risk screening leading to potential cost savings and better quality of life when compared with the current screening methods for T2D risk.

There is an urgent need for health care systems to find new ways to prevent and treat diseases effectively and cost-efficiently. Using existing genomic data from biobanks can be seen as a possibility to develop new solutions at the level of populations as presented in this Research Topic. Biobank donors appeared to accept the approaches described and some health benefit was observed. Much research is still needed on the utility of genomic results to the donors and the health care in the long run.

